# GaAs/GaP Superlattice Nanowires for Tailoring Phononic Properties at the Nanoscale: Implications for Thermal Engineering

**DOI:** 10.1021/acsanm.3c04245

**Published:** 2023-10-05

**Authors:** Aswathi K. Sivan, Begoña Abad, Tommaso Albrigi, Omer Arif, Johannes Trautvetter, Alicia Ruiz Caridad, Chaitanya Arya, Valentina Zannier, Lucia Sorba, Riccardo Rurali, Ilaria Zardo

**Affiliations:** † Department of Physics, University of Basel, 4056 Basel, Switzerland; ‡ Institut de Ciència de Materials de Barcelona, ICMAB-CSIC, Campus UAB, 08193 Bellaterra, Spain; § NEST, Istituto Nanoscienze-CNR and Scuola Normale Superiore, 56127 Pisa, Italy; ∥ Swiss Nanoscience Institute, University of Basel, 4056 Basel, Switzerland

**Keywords:** phonons, superlattices, nanowires, metamaterials, Raman spectroscopy, Brillouin interferometry

## Abstract

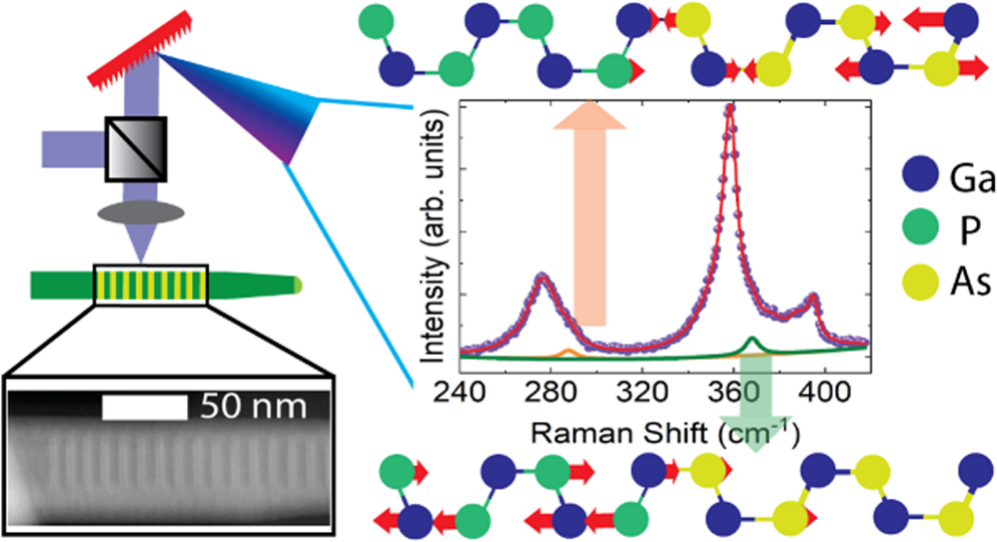

The possibility to tune the functional properties of nanomaterials is key to their technological applications. Superlattices, i.e., periodic repetitions of two or more materials in one or more dimensions, are being explored for their potential as materials with tailor-made properties. Meanwhile, nanowires offer a myriad of possibilities to engineer systems at the nanoscale, as well as to combine materials that cannot be put together in conventional heterostructures due to the lattice mismatch. In this work, we investigate GaAs/GaP superlattices embedded in GaP nanowires and demonstrate the tunability of their phononic and optoelectronic properties by inelastic light scattering experiments corroborated by ab initio calculations. We observe clear modifications in the dispersion relation for both acoustic and optical phonons in the superlattices nanowires. We find that by controlling the superlattice periodicity, we can achieve tunability of the phonon frequencies. We also performed wavelength-dependent Raman microscopy on GaAs/GaP superlattice nanowires, and our results indicate a reduction in the electronic bandgap in the superlattice compared to the bulk counterpart. All of our experimental results are rationalized with the help of ab initio density functional perturbation theory (DFPT) calculations. This work sheds fresh insights into how material engineering at the nanoscale can tailor phonon dispersion and open pathways for thermal engineering.

## 1 Introduction

In the last few decades, technological miniaturization has led to the creation of smaller and more efficient electronic devices. As devices get smaller, we increasingly face the need for efficient heat management at lower dimensions, typically at the nanoscale.^1,2^ Designing materials with tailorable thermal properties is very attractive in this regard.^3,4^ The thermal conductivity of materials quantifies the efficiency at which heat energy is transferred within them. Consequently, the capability to tune the thermal conductivity becomes essential for effective thermal engineering. Depending on specific applications, there is a necessity to engineer materials with heightened thermal conductivities, such as in high-power electronics^5^ and computing devices, as well as materials with reduced thermal conductivities for use in thermoelectric applications,^6^ thermal barrier coatings,^7^ etc. In semiconducting and dielectric materials, phonons - the vibrations of the atomic lattice - are responsible for heat and sound transport.^8^ From kinetic theory, the thermal conductivity in semiconductors depends on the phononic properties such as density of states, phonon mean free path, group velocities, and phonon energies.^9,10^ Consequently, the deliberate manipulation of these phononic properties enables us to create materials with tailor-made thermal conductivity. The advancements in material growth techniques have enabled the growth of artificial material structures with tunable phonon spectra for thermal engineering.^3,11−18^ Among the various strategies, the utilization of superlattice (SL) structures emerges as a promising way to tune the thermal property of materials.^11,19,20^ SLs are compositionally modulated periodic structures and have been shown to host a plethora of interesting physics.^11,21^ Since the first experimental observation of reduced thermal conductivity in AlAs/GaAs SLs,^22^ several theoretical and experimental works have explored SL structures for their potential applications in thermoelectric devices. The theoretical work of Hyldgaard and Mahan^23^ describes a model to calculate the SL thermal conductivity based on modifications to the SL phonon spectrum. They predicted a reduction in the thermal conductivity of Si/Ge SLs due to the decrease in the group velocity of the SL phonon modes. This model was further extended by Tamura et al.,^24^ who calculated phonon group velocities in both Si/Ge and AlAs/GaAs SLs and showed their contributions to thermal conductivity.

SLs with atomically clean interfaces have proven to be ideal systems for studying the crossover from particle to wave nature of phonons.^10,25^ Indeed, several studies have demonstrated that this crossover can be obtained by controlling the SL periodicity, showing that a distinctive minimum in the lattice thermal conductivity can be achieved.^11,26,27^ By taking advantage of wave interference phenomena of phonons, SLs can also serve as an ideal platform for creating phonon bandgap materials by selectively allowing (blocking) phonons with certain energies to propagate through them. Moreover, studies have shown that we can have control over coherent and incoherent phonon transport in SLs by means of band folding produced by the larger periodicity of the SL and the related changes in the phonon density of states and velocities.^10,11,17,20^ The understanding of particle-wave duality of fundamental excitations like electrons and photons has revolutionized modern electronics and optics. By extension, a similar understanding of phonons is highly desirable for designing new ways for manipulating the heat flow in materials. However, the need for nanoscale periodicity and atomically smooth interfaces heavily constrains the design of the SL for coherent control of phonon transport.

SL structures can take different forms depending on their constituent layers. Conventional SL structures are formed by the periodic alternation of different materials.^28−30^ However, SLs can also be made by periodic alternation of different crystal phases of the same material^31^ and by the periodic rotation of the crystal lattice, the so-called twinning SLs.^17,32^ The growth of many of these superstructures with atomically abrupt interfaces or minimal interface mixing, however, is limited to thin films. One of the main issues is the lattice mismatch between the constituent layers. Nanowires (NWs) are quasi-one-dimensional structures which allow the growth of defect-free axial and radial heterostructures otherwise difficult or even impossible to achieve in planar structures because they can release the strain caused by the lattice mismatch in the radial direction.^33−35^ Advancements in the epitaxial growth of semiconductor NWs make it possible to grow SL NWs of different types with atomic interface sharpness and minimal interface mixing. This makes SL NWs an ideal platform to envisage and study phonon engineering at the nanoscale for controlled heat transport. In recent years, there has been an increase in both the theoretical and experimental studies of SL NWs with a focus on their thermal properties.^17,36−38^ Most of the work done in NW SLs from the perspective of phonon engineering explores twinning or crystal phase SLs. In this work, we study the phononic properties of SL NWs made of two different constituent materials: GaAs and GaP. The combination of materials with different elastic constants creates an enhancement of phonon wave interference effects such as phonon bandgaps.^16^ Both GaAs and GaP are highly relevant materials in the realm of photonic and optoelectronic devices with applications in photovoltaics,^39−41^ light-emitting devices,^42^ transistors,^43^ etc. The possibility of combining these two materials at the nanoscale will create a versatile material system for photonic and phononic applications. However, the lattice mismatch between GaAs and GaP is approximately 3.7%, posing growth constraints for planar superlattices.^21^ The NW geometry enables the combination of GaP and GaAs epitaxially without the formation of misfit dislocations at the interface.

In this work, we investigate the phononic properties of GaAs/GaP SL NWs with periodicities ranging from 4.8 to 10 nm. We present the results of inelastic light scattering experiments such as Raman microscopy and Brillouin light scattering interferometry of these SL NWs, and we show that the phononic properties of GaAs/GaP SL NWs can be tuned by controlling the SL periodicity. We demonstrate that as the SL periodicity increases, the number of phonon modes increases. Our experimental results together with the ab initio calculations indicate a reduction in the electronic bandgap in the SL structure compared to the bulk materials. Our study shows that NWs can serve as a template for combining different materials at the nanoscale for designing new material systems with tunable phononic and optoelectronic properties.

## 2 Results and Discussion

**Figure 1:**
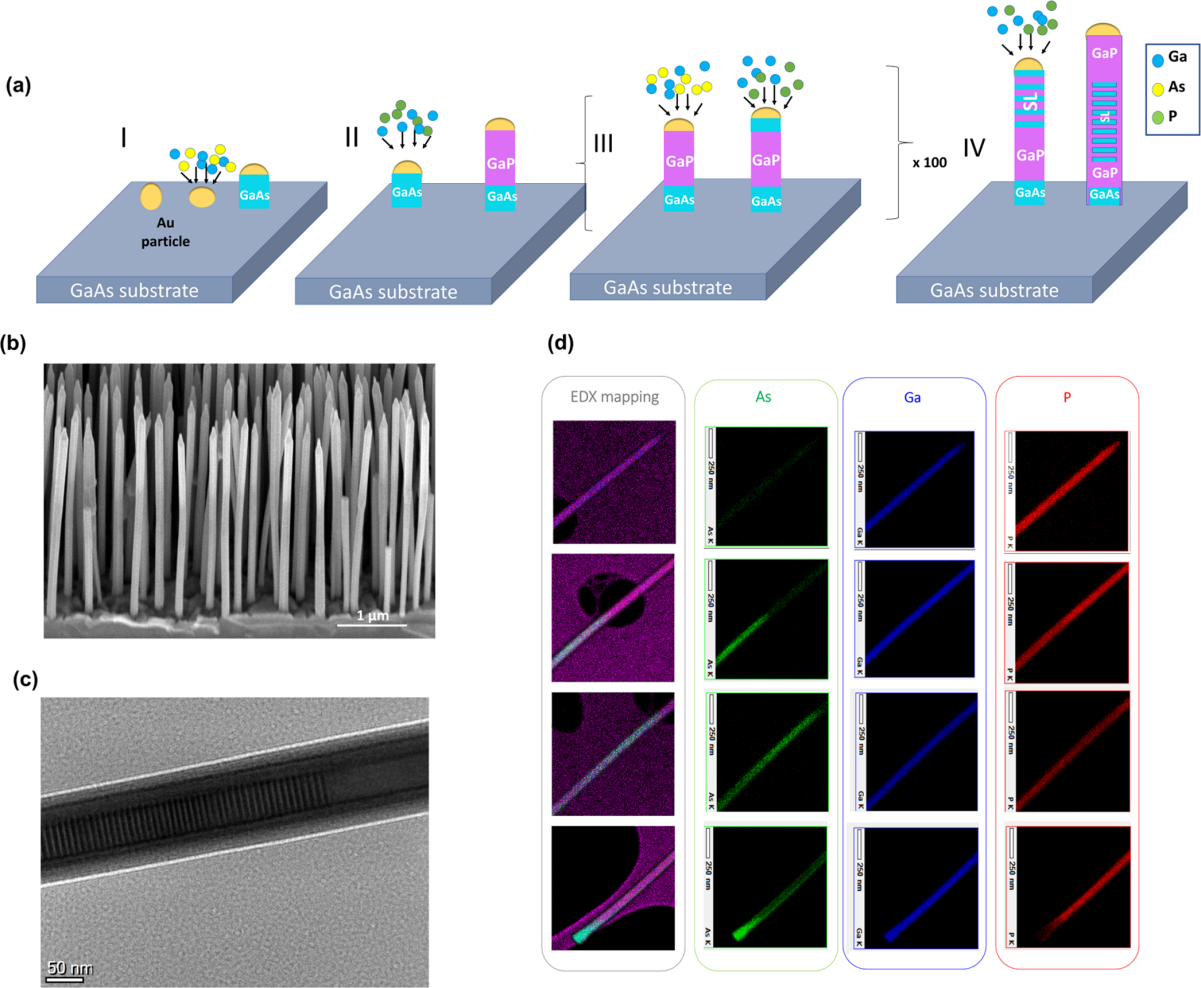
GaAs/GaP superlattice nanowires. (a) Schematic illustration of the four-step growth process of a typical GaAs/GaP SL NW using chemical beam epitaxy; (b) SEM image of as-grown free-standing GaAs/GaP SL NWs grown on GaAs substrate; (c) TEM image of a typical SL NW of a nominal period 10 nm dispersed in a holey carbon grid investigated in this work; (d) the EDX mapping of SL NW with 4.8 nm period in false colors shows the distribution of elements along the length of the wire showing the presence of the GaAs/GaP SL segment. The elemental maps of one NW are represented as follows: green for As, blue for Ga, and red for P.

GaAs/GaP SL NWs with different periodicities are grown using Au-assisted chemical beam epitaxy (CBE) on GaAs (111) B substrates. The GaAs/GaP SL NW growth process is described in detail elsewhere.^44^Figure 1(a) illustrates the four-step Au-catalyzed epitaxial growth process of a typical GaAs/GaP SL NW using CBE. Figure 1(b) shows a scanning electron microscopy (SEM) image of a representative as-grown sample of GaAs/GaP SL NWs with relatively homogeneous lengths (3.58 ± 0.24 μm) and diameters (106 ± 10 nm). The structural and chemical composition of the GaAs/GaP SL NWs were studied using a transmission electron microscope (TEM) and energy-dispersive X-ray analysis (EDX) measurements. The period L of the SL structure is the sum of the lengths of consecutive GaAs (*L*_GaAs_) and GaP (*L*_GaP_) segments, i.e., *L* = *L*_GaAs_ + *L*_GaP_. Figure 1(c) shows the TEM image of a SL NW with a nominal period of 10.0 nm (*L*_GaAs_ = 5.0 nm and *L*_GaP_ = 5.0 nm). The detailed high-resolution TEM (HR-TEM) image of an SL NW with a 4.8 nm long period is found in the SI in Figure S1. The TEM image shows alternatingly spaced GaAs and GaP segments along the growth direction of the NW and from the HR-TEM in Figure S1, we measure L as 5.3 ± 0.6 nm, i.e., consistent with the nominal value, using DigitalMicrograph. Figure 1(d) shows the EDX elemental mapping of a whole NW, in 4 different positions along the growth axis, from the base (bottom panel) to the tip (top panel). The EDX elements are represented in false colors as follows: As in green, Ga in blue, and P in red. The NW is mapped from bottom to top as follows: at the bottom of the wire, we have a first segment composed of GaAs, this is shown in the EDX map in the color combination of blue and green as cyan in Figure 1(d). After this segment, we have a long GaP segment, shown in the color combination of red and blue as magenta, which is followed by the SL segment, depicted in Figure 1(d) as a combination of red, blue, and green. The SL segment consists of approximately 100 repetitions of the GaAs + GaP unit. At the top part of the NW, we have a final section of the GaP segment shown again in the false color combination of red and blue in Figure 1(d). The other samples of this work have the same design and compositional sequence, they differ only for the nominal SL periodicity: *L* = 6.0 nm (*L*_GaAs_ = 3.0 nm and *L*_GaP_ = 3.0 nm) and 10.0 nm (*L*_GaAs_ = 5.0 nm and *L*_GaP_ = 5.0 nm). A reference sample of NWs without SL, i.e., made of a 0.5 μm long GaAs stem followed by 3 μm of GaP segment, was also grown.

In order to probe the phonon spectra of GaAs/GaP SL NWs, we performed inelastic light scattering experiments such as Raman microscopy on single GaAs/GaP SL NWs with different period lengths and Brillouin light scattering interferometry on the as-grown NW ensemble.

**Figure 2:**
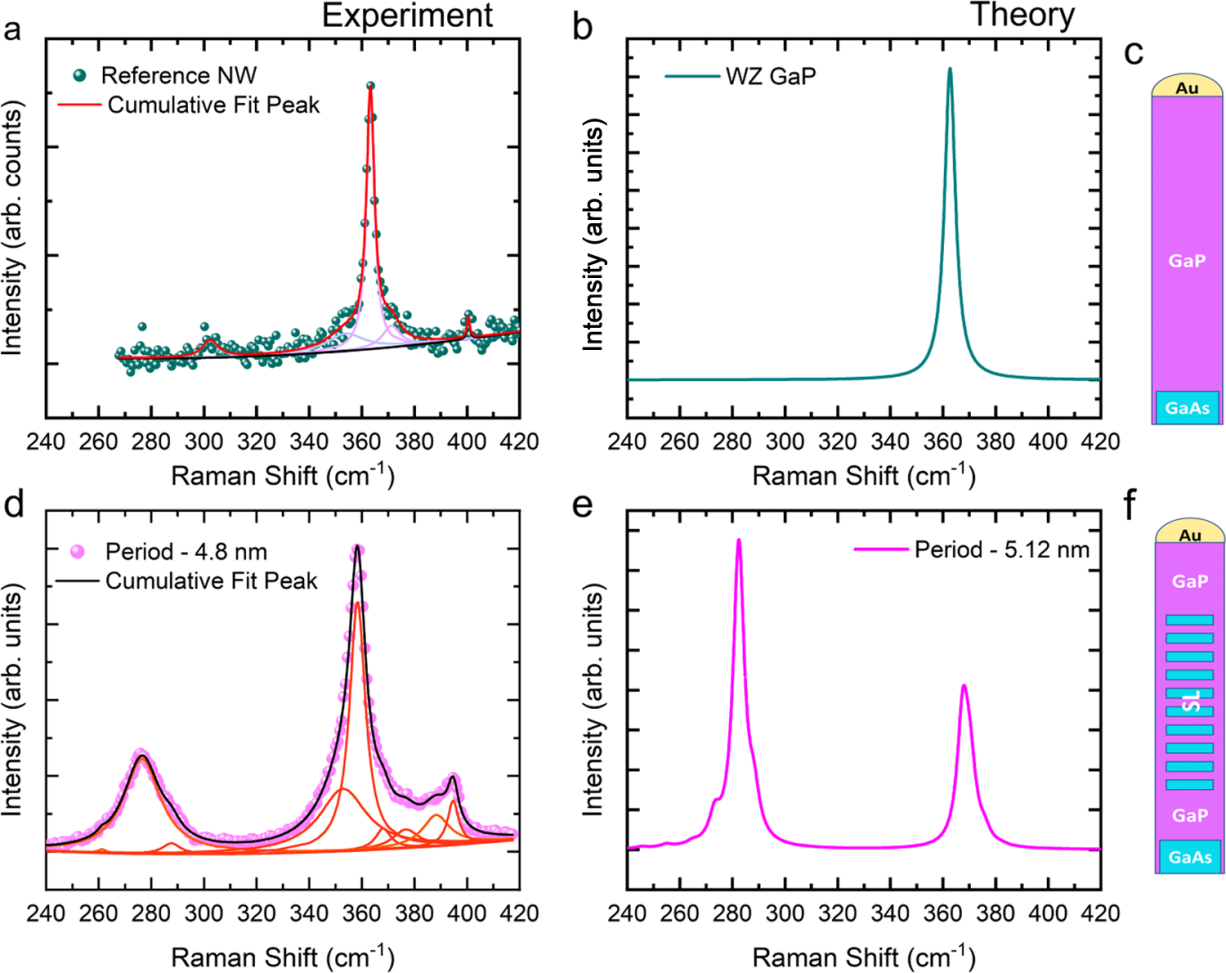
(a) Measured spectrum of GaP from reference NW with the experimental data plotted in green spheres, the cumulative Lorentzian fit is shown in solid red line, the deconvoluted Lorentzian fits are shown in violet curves, and the baseline in black. (b) Calculated spectrum of bulk wurtzite GaP. (c) Schematic of the GaP reference NW from which the spectrum in (a) is obtained. (d) Measured spectrum of a GaAs/GaP SL NW with a period of 4.8 nm with the experimental data shown in magenta spheres and the cumulative Lorentzian fitting in solid black curve; red curves show the deconvoluted Lorentzian fits, and the orange line shows the baseline. (e) Calculated Raman spectrum of a GaAs/GaP SL NW with a period of 5.12 nm. (f) Schematic of the GaAs/GaP SL NW from which the spectrum in (d) is taken.

In Figure 2(a), we display the experimental Raman spectrum obtained from the GaP reference NW in the *x̅*(*z*,*z*)*x* scattering configuration in Porto notation, as described in Section 4. The experimental data are plotted with dark green spheres, and the solid lines correspond to the Lorentzian fitting. The red solid curve corresponds to the cumulative peak fitting, while the purple curves correspond to the deconvoluted Lorentzian used to fit the individual peaks. Between 340 and 410 cm^–1^, the experimental data can be fitted with 4 peaks at frequencies 352.2, 362.2, 371.0, and 400.7 cm^–1^, which can be assigned to the *E*_2_^H^ mode, the *A*_1_/*E*_1_ transversal optical (TO) mode, the surface optical (SO) mode, and the longitudinal optical (LO) mode, respectively.^45−47^ The calculated spectrum of bulk wurtzite (WZ) GaP for configuration *x̅*(*z*,*z*)*x* is shown in Figure 2(b). We observe a peak at 362.6 cm^–1^ which corresponds to the TO mode of GaP in good agreement with the experimental observation. We observe a low-intensity LO mode in the experimental spectrum, in principle forbidden, and thus absent in the calculated spectrum. This observation is due to the usage of a high numerical aperture (NA) objective and due to the partial relaxation of the selection rules, similar to what is observed and explained in De Luca et al.^17^ Similarly, we observe a peak around the frequency of the *E*_2_^H^ mode, a signature of the WZ crystal phase. The *E*_2_^H^ mode is also forbidden in the *x̅*(*z*,*z*)*x* configuration, and its observation can also be assigned to the use of a high NA objective as well as due to the small deviations in the selection rules. It is worth noticing that, it has been shown that the *E*_2_^H^ has a strong wavelength dependence.^48^ Also, we cannot exclude that the signal we observe is due to an asymmetric broadening of the GaP TO mode because of the anharmonicity of the material.^49^ In Figure S2, we have presented the results of the Raman spectroscopy done in the *x̅*(*y*,*y*)*x* configuration for the reference NW where the presence of the *E*_2_^H^, which is allowed in this scattering geometry, can be better appreciated.^17,50,51^ The SO, on the other hand, is due to finite size effects because of the NW geometry,^45,52^ and its frequency depends mainly on the dielectric constant of the surrounding medium as well as on the diameter of the NW. Therefore, it is absent in the computed spectrum, as the calculations were performed in a bulk system. In Figure 2(a), we also see a small peak at around 300 cm ^–1^ arising from the Si substrate beneath.^53^Figure 2(c) is a schematic of the reference NW from which the spectrum in Figure 2(a) was obtained.

In Figure 2(d), we display the spectrum obtained experimentally from a 4.8 nm period GaAs/GaP SL NW (whose schematic is depicted in Figure 2(f)) in the *x̅*(*z*,*z*)*x* configuration, probing the region of the NW embedding the SL. In the spectral region of the phonon modes of GaP, we observe several modes between 340 and 400 cm^–1^. The most intense peaks in this spectral range are at frequencies of 358.3, 352.8, and 388.2 cm^–1^ in the order of decreasing intensities. These new peaks appearing are assigned to modes arising from the SL. We can also see several Raman peaks in the spectral region (between 240 and 300 cm^–1^) close to the phonon modes of GaAs, the most intense peak being around 276.4 cm^–1^. The other peaks are located at 261.3 and 287.6 cm^–1^. In Figure 2(e), we present the calculated spectrum for a GaAs/GaP SL with a period of 5.12 nm. In the calculated spectrum, we have fixed the full width at half-maximum (FWHM) of the peaks at 5 cm^–1^, since it is similar to the experimentally measured peak broadening. The theoretical spectrum of the SL structure contains several peaks between 240 and 300 cm^–1^ as well as between 340 and 400 cm^–1^ (not distinguishable in the convoluted spectrum with the given FWHM; a list of the computed Γ-point frequencies, setting a threshold intensity indicative of their experimental detectivity, is given in Table S1). Experimental and theoretical spectra obtained from an SL with a longer period (10.0 and 6.39 nm, respectively) are shown in Figure S3 in the Supporting Information.

These several phonon modes can be understood in terms of backfolding of the phonon dispersion of the constituent materials of the SL or, equivalently, from the fact that the SL period dictates a much larger unit cell, containing many more atoms than the WZ primitive cells of the constituent materials.^54^ The phonon dispersion of the GaP/GaAs SL with a period of 6.39 nm obtained through ab initio calculations can be found in Figure S4 in the Supporting Information. There, the increased number of phonon modes at the Γ-point can be appreciated, though it is worth noticing that not all of them are Raman-active (Table S1).

Furthermore, to prove the origin of the phonon modes in the two different frequency ranges (i.e., 240–300 and 340–400 cm^–1^), we then analyzed the corresponding eigen-displacements. Figure S5(a,b) in the SI shows the schematic for two phonon modes at 288.1 and 367.2 cm^–1^, respectively, selected from Table S1. In the schematic, the blue, yellow, and green spheres represent Ga, As, and P atoms, respectively. The phonon mode at 288.1 cm^–1^ involves vibrations of Ga and As atoms while the phonon mode at 367.2 cm^–1^ involves vibrations of the Ga and P atoms. Therefore, for the sake of simplicity, from now on, we will refer to SL modes in the low-frequency range as to GaAs-like SL mode and to more in the high-frequency range as to GaP-like SL modes. For the sake of generality, in Figure S6 in the Supporting Information, we also show the eigen displacement of a Raman-inactive, low-frequency mode, whose vibrations involve atoms on both the GaP and the GaAs region.

**Figure 3:**
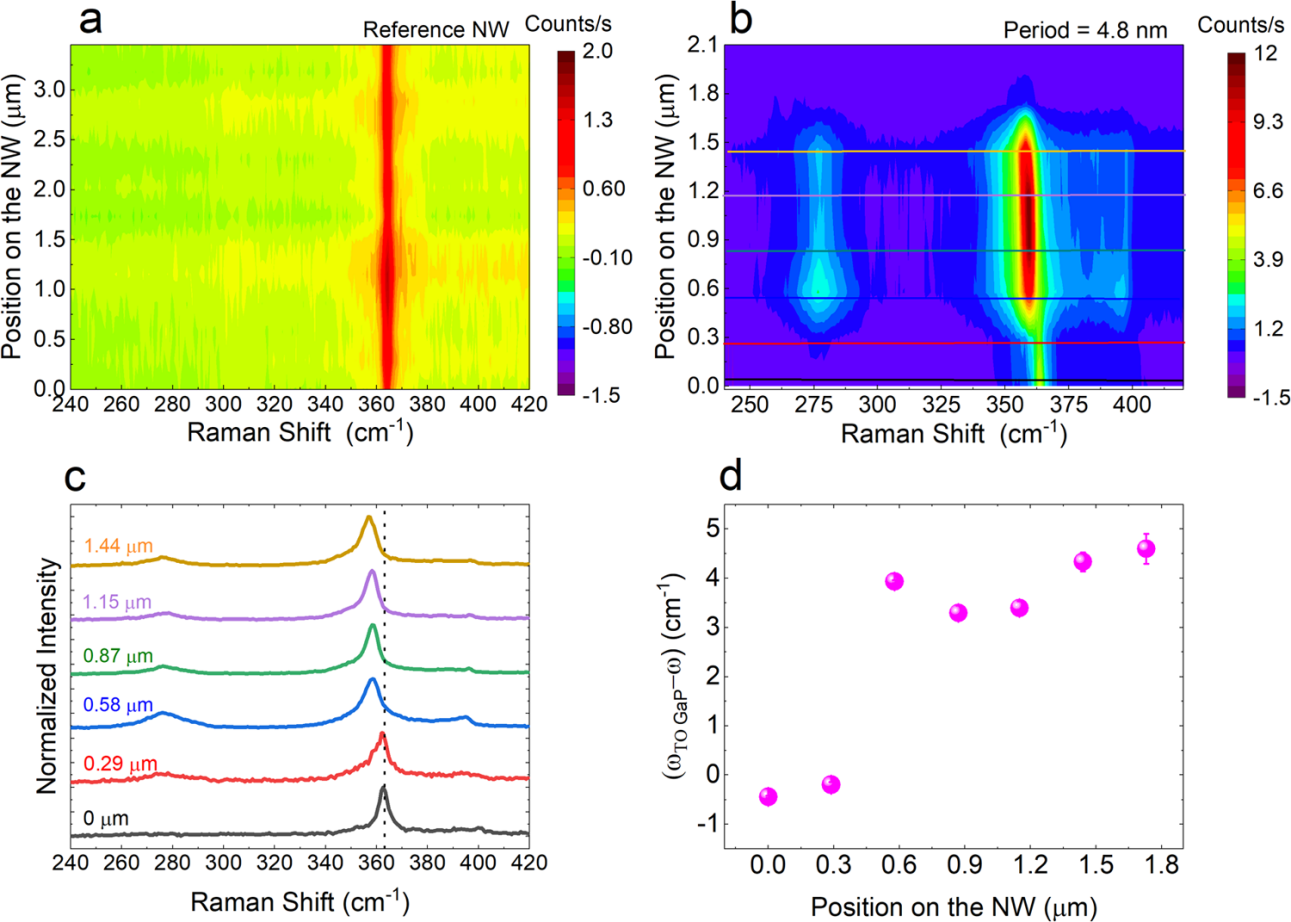
Spatially resolved Raman map of (a) GaP reference NW; (b) GaAs/GaP SL NW of period 4.8 nm; (c) individual spectra corresponding to the horizontal lines extracted from (b); (d) relative shift of the mode highest-intensity mode in the GaP-like region with respect to the frequency of the experimental GaP TO mode as a function of the position along the NW.

We also performed spatially resolved one-dimensional μ-Raman measurements along the length of the GaAs/GaP SL NW of the period 4.8 nm as well as that of the GaP reference NW studied in Figure 2. We collected Raman spectra every 300 nm since the spatial resolution is also limited by our spot size, which is around 420 nm. In Figure 3(a), the result of the Raman scan of the GaP reference NW is plotted in a two-dimensional (2D) false colormap with the *x*-axis showing the Raman shift and the *y*-axis showing the laser position along the NW. From the map, we see only signal arising from GaP, i.e., the TO mode at ∼362 cm^–1^. In Figure 3(b), we present the results of a similar scan performed on the GaAs/GaP SL NW of the period 4.8 nm shown in Figure 2(d). In the map, we see the signal from GaP, followed by SL signal for about 1 μm, and a weak GaP signal after the SL segment. We do not see any signal arising from the GaAs segment in the reference NW or the SL NW. This could possibly be due to breaking of the NW at the junction between GaAs and GaP during the transfer using a micromanipulator. In Figure 3(c), we present individual spectra extracted from Figure 3(b). The black dotted line is a guide for the eye to observe the downward shift in the frequency of the high-intensity peak from the GaP-like modes. In Figure 3(d), the shift of the highest-intensity GaP-like SL peak from the TO of WZ GaP measured in the reference NW is shown as a function of the position along the NW. Outside the SL, the frequency of the highest-intensity peak in the GaP-like modes coincides with that of the WZ GaP TO mode. However, on entering the SL segment, the frequency of the highest intensity down-shifts considerably. This is a further indication of the different origins of these modes: the SL acts like a different material system with its own characteristic phonon modes.

After observing the appearance of the SL modes in the 4.8 nm period GaAs/GaP SL NW, we tested the tunability of the phonon modes by varying the SL period length. In Table 1, we present the calculated Raman-active phonon modes at the Γ point for three different periods; the threshold intensities are set according to their experimental detectability like in Table S1. From Table 1, we see that as the period increases, the number of phonon modes also increases. Similarly, we also see that different periods have distinct phonon modes. We have performed both Brillouin and Raman scattering experiments on NWs with different SL periods to understand the effect of SL periodicity on the acoustic and optical phonons, respectively.

**Table 1:** Calculated Frequencies of Raman-Active Phonon Modes for a GaAs/GaP SL Structure with 3 Different Periods^a^

frequencies for *L* = 3.83 nm (cm^–1^)	frequencies for *L* = 5.12 nm (cm^–1^)	frequencies for *L* = 6.39 nm (cm^–1^)
247.53	245.55	243.87
259.88	254.87	250.83
272.02	264.73	259.57
279.57	273.24	267.39
285.13	281.13	274.60
366.94	282.45	279.78
368.35	288.12	283.64
371.79	367.25	285.71
379.98	368.09	290.04
	370.23	367.33
	372.06	367.86
	375.85	369.49
	382.31	370.64
		373.38
		378.84

a Phonon modes with a Raman intensity more than 100 times smaller than that of the most intense mode were considered nondetectable.

**Figure 4:**
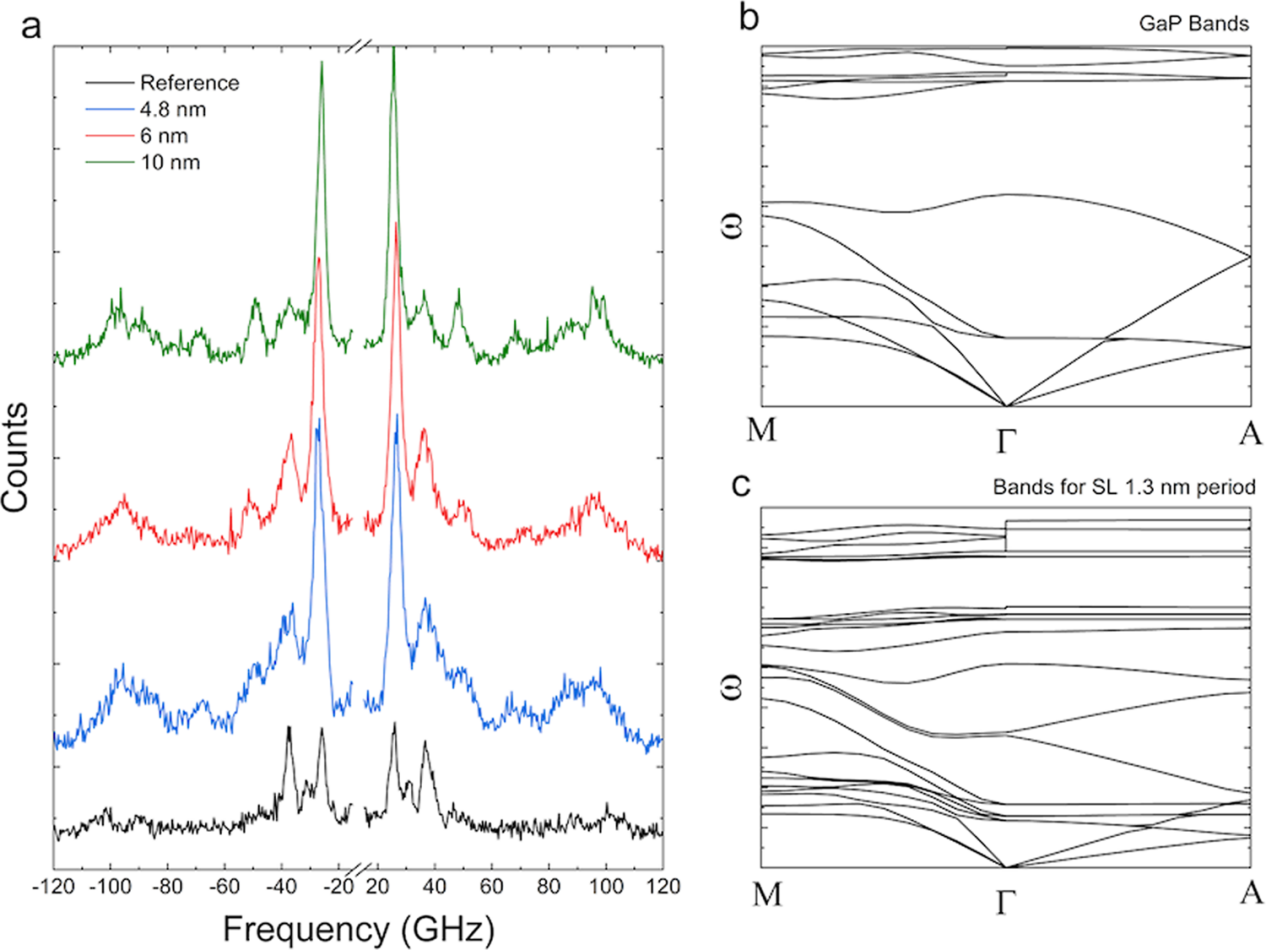
(a) Brillouin scattering spectrum of GaAs/GaP SL NWs of different periods. the curve in black represents the signal from the GaP reference, while the curves in blue, red, and green are collected from 4.8, 6, and 10 nm periods SLs, respectively. (b) Calculated band structure of bulk WZ GaP. (c) Calculated band structure of an SL of 1.3 nm period.

**Figure 5:**
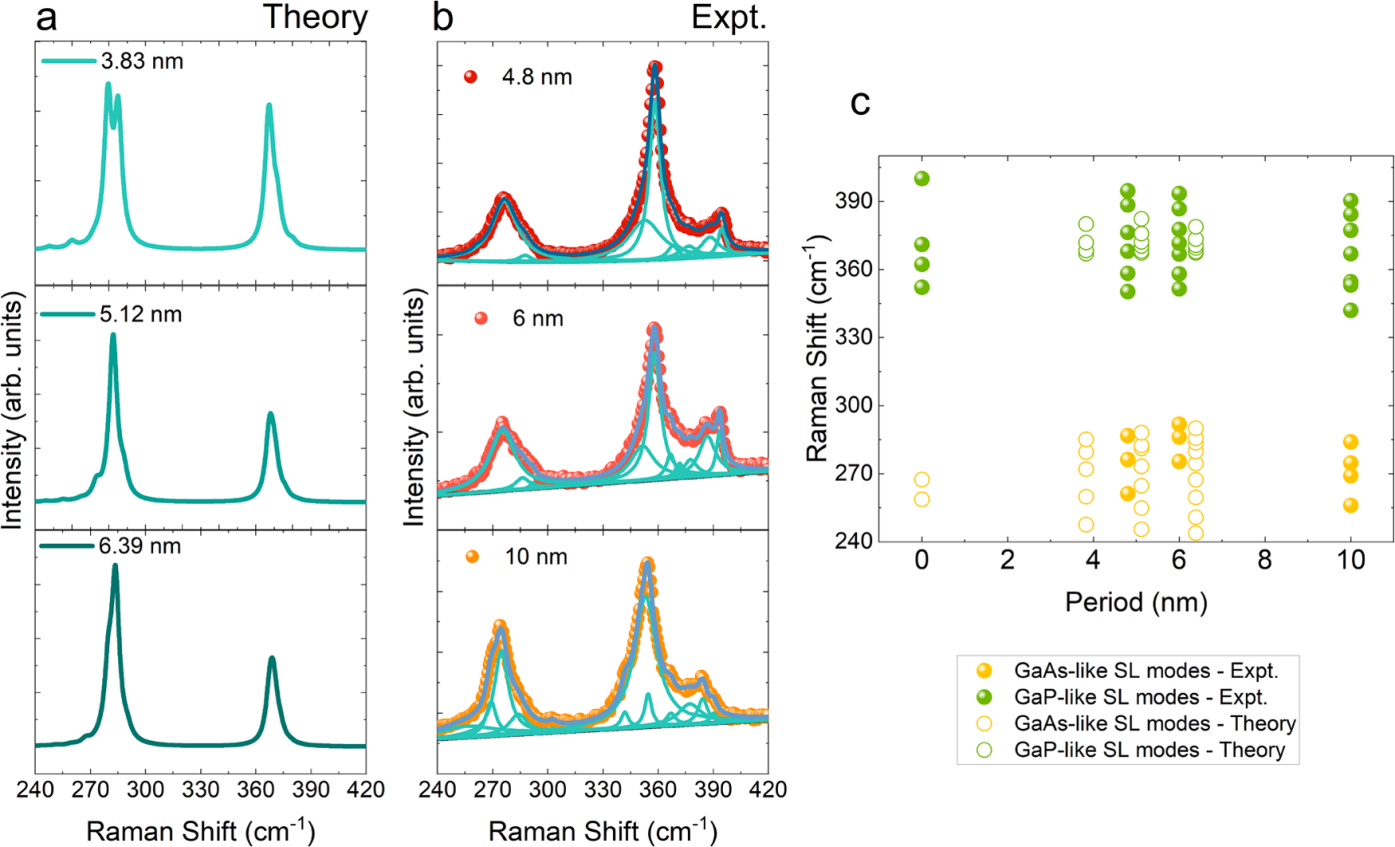
(a) Calculated Raman spectra of 3 different SL periods. (b) Experimental Raman spectra obtained from 3 different SL periods along with the deconvoluted Lorentz fitting. (c) Raman peak positions extracted from both experimental (filled circles) and calculated (open circles) Raman spectra as a function of period length.

The acoustic phonon modes were probed by Brillouin light scattering (BLS) interferometry.^55,56^Figure 4(a) shows the BLS spectra of SL NWs with different periods as well as that of the reference NW. The BLS spectra were fitted with Lorentzian curves to extract the peak position of the signal. From the reference sample, we fit 4 peaks, centered around 25.6, 30.8, 37.1, and 101.3 GHz, while for an SL with *L*= 10 nm, we extracted 8 peaks centered around 25.4, 29.2, 36.1, 48.3, 68.1, 90.7, 95.43, and 99 GHz. For the SL sample with SL period 6 nm, we fit the spectrum with 5 curves centered around, 26.4, 36.6, 49.6, 72.1, and 95.6 GHz. And for an SL with *L* = 4.8 nm, we fit the data with 7 peaks centered around 26.4, 36.1, 40.5, 50.1, 68.5, 86.1, and 97 GHz. From our experimental results, we observe a greater number of phonon modes in the SL NWs compared to the reference NW. To understand the modifications in the acoustic phonons in the SL structure, we computed phonon dispersion of WZ GaP as well as GaAs/GaP SLs of different periods. In Figure 5(b,c), we show the computed band structure of WZ GaP and a GaAs/GaP SL with *L* = 1.3 nm, respectively. We can see that compared to the GaP bands, the 1.3 nm long period has a greater number of phonon bands in the dispersion. Dispersions of an SL with *L* = 6.39 nm are shown in Figure S4. In our BLS experiments, we use a laser line at 532 nm for the excitation, the wave vector is given by *q* = 4πn/λ nm^–1^, where n is the refractive index.^57^ In our case, we get the q value at around 85 μm^–1^. From the calculations done for bulk GaAs/GaP SL with periodicity 6.39 nm, within our experimental detection window (<120 GHz), there are two modes at around 35 GHz and around 75 GHz. In our experiments, for the GaAs/GaP SL NW with *L* = 6 nm, we see peaks around 36 and 71 GHz, in very good agreement with the calculation. Within our experimental detection window, i.e., for phonon frequencies between −120 and 120 GHz, for q values close to 85 μm^–1^, we obtain a good agreement with calculation. However, we also observe a few phonon modes which do not appear in the calculations. This deviation between calculation and experiment could be explained as due to a combination of several reasons, the first is the difference between experimentally measured and calculated phonon frequencies leading to calculated modes falling out of the experimental detection window. In our calculations of the dispersion relationship, we do not take into account the surface phonon modes arising from the surface ripple mechanism,^58,59^ which is often observed in Brillouin interferometry of metals and semiconductors. Our bulk calculations also do not take into account confined acoustic phonon modes reported, e.g., by Kargar and co-workers in GaAs NWs with diameters in 100–150 nm range.^57^ We have tabulated the calculated acoustic phonon modes with frequencies less than 1 THz for an SL with periodicity of 6.3 nm in the SI in Table S2.

**Figure 6:**
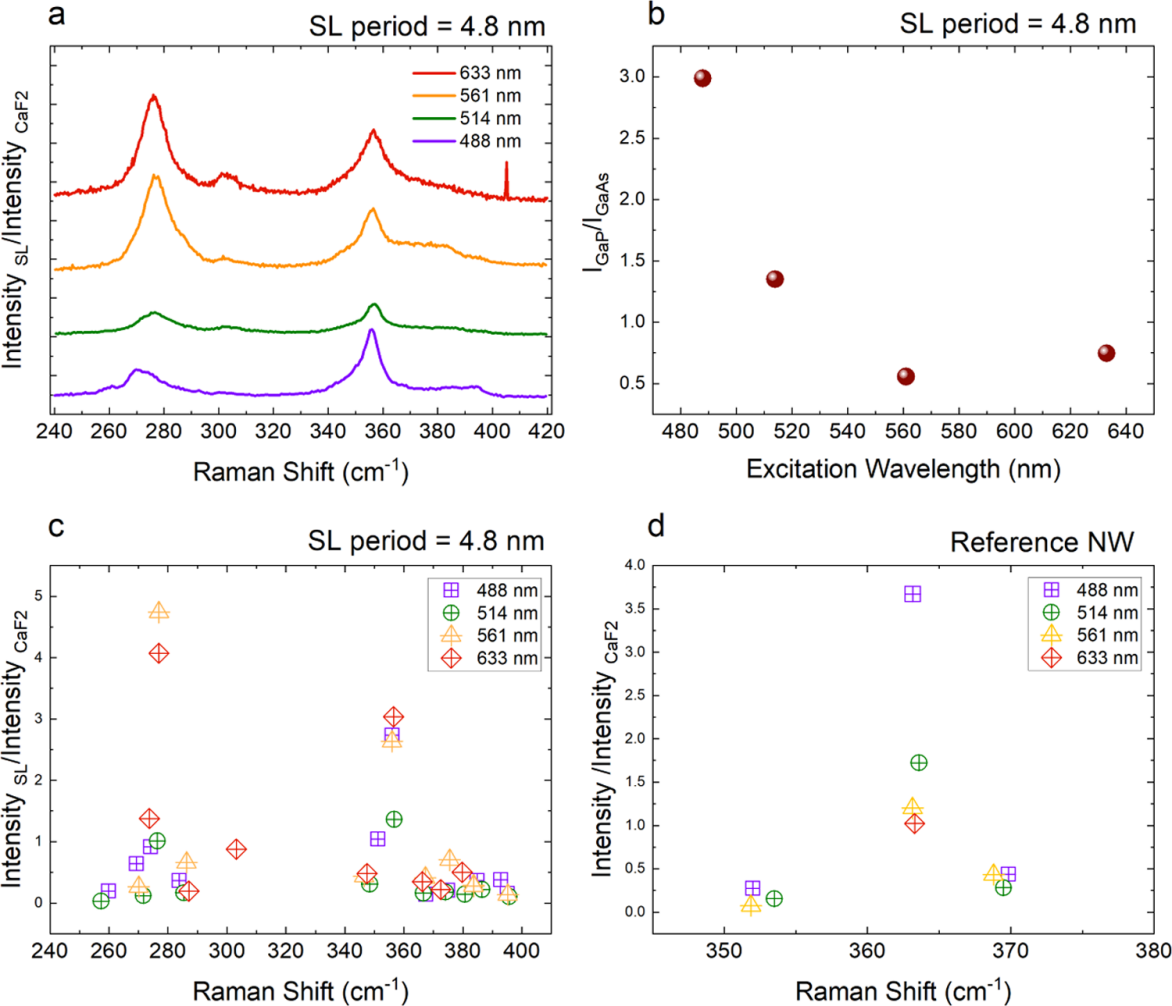
(a) *y*-Offset plots of Raman spectra of a 4.8 nm period GaAs/GaP SL NW obtained with 488 nm(violet), 514 nm (green), 561 nm (orange), and 633 nm (red). The individual spectra are normalized using CaF_2_ Raman intensity for the given excitation wavelength; (b) ratio of intensity of GaP-like mode around 356 cm^–1^ to intensity of GaAs-like mode around 276 cm^–1^ as a function of excitation wavelength; (c) intensities (normalized with CaF_2_ intensity) of different phonon modes as a function of the Raman shift extracted from (a) with same color scheme as in (a); (d) intensities (normalized with CaF_2_ intensity) of different phonon modes as a function of the Raman shift for a reference NW, with same color scheme as in (a).

Figure 5 presents the overview of the results on tunability of the optical SL phonon modes as a function of SL periodicity for GaAs and GaP SL NWs. In Figure 5(a), the calculated Raman spectra obtained for different SL periodicities (3.83–6.39 nm) are shown as solid lines, while in Figure 5(b), we present the μ-Raman spectra taken from SLs of different periodicities (4.8–10 nm period). The experimental data are represented by colored spheres, while the solid curves show the Lorentz curves used for fitting. The quantitative analysis of the SL modes is summarized in Figure 5(c). Open circles represent the peak positions extracted from calculated spectra, while filled circles represent the peak positions extracted from Lorentzian fits of the experimental Raman measurements. The colors yellow and green are used to denote GaAs- and GaP-like SL modes, respectively. We also observe, both in the experimental and in the calculated spectra, that as the periodicity increases, the number of Raman-active phonon modes also increases, as is expected due to the increased number of atoms in the unit cell of the periodic superstructure and the related backfolding of the phonon dispersion. One major difference between the calculated and experimental spectra is the relative intensity between the GaAs-like and GaP-like modes. For calculated spectra, the intensity of GaAs-like modes is higher than that of GaP-like modes, while for experiments performed using a 488 nm laser, the GaP-like modes are more intense. This is possibly due to resonant conditions. Namely, the SL exhibits also a modified electronic band structure, whose details (e.g., number of bands, their dispersion, and energy gaps) depend on the periodicity of the SL. Therefore, we performed wavelength-dependent μ-Raman measurements on a GaAs/GaP SL NW with *L* = 4.8 nm to test the effects of the excitation wavelength on the SL phonon modes. The results of wavelength-dependent measurements are summarized in Figure 6. In our wavelength-dependent Raman studies, we use CaF_2_ as the reference material as it has a constant Raman tensor in the range 1.8–3.8 eV.^60,61^ We first normalized all of the measured spectra with the intensity of the CaF_2_. In Figure 6(a), we present the normalized results of Raman measurements from an SL NW with *L* = 4.8 nm excited with 3 different wavelengths. The data in violet, green, orange, and red curves represent spectra obtained with excitation wavelengths 488, 514, 561, and 633 nm, respectively. One striking observation as we increase the excitation wavelength is that the relative contribution of GaAs-like modes compared to GaP-like modes increases. This is clearer in Figure 6(b), shown as the ratios of intensities of the most intense peak for the GaP-like mode (at around 356 cm^–1^) to the intensity of the most intense GaAs-like mode (at around 276 cm^–1^). We see that the value of this ratio goes from around 3 to less than 0.5 as we increase the excitation wavelength from 488 to 561 nm, this value then slightly increases to 0.7 at 633 nm. In Figure 6(c), we plot the intensities of the GaAs-like and GaP-like modes the violet, green, orange, and red symbols represent excitation wavelengths 488 nm (2.54 eV), 514 nm (2.41 eV), 561 nm (2.21 eV), and 633 nm (1.95 eV), respectively. For the SL NW excited with different wavelengths, we see that GaAs-like phonon mode around 276 cm^–1^ shows a significant increase in signal at 561 and 633 nm. For GaP-like phonon modes, the phonon mode around 356 cm^–1^ shows comparable intensities for 633, 561, and 488 nm and a lower intensity for 514 nm. All other SL phonon modes presented comparable intensities at different excitation wavelengths. In Figure 6(d), we present the results of similar measurements done on the reference NW. For the WZ GaP signal of the reference NW, we see that the signal intensity decreases for increasing excitation wavelength, with the highest intensity for 488 nm, followed by 514 nm, then by 561 nm and lowest intensity for 633 nm. This trend in intensities points to approaching resonant conditions as we increase excitation energy. It is worth pointing to a previous work done using resonant Raman studies to extract the electronic band structure of WZ GaP NW bandgap which has shown that E_1_(LO) mode shows resonances at 2.38 and 2.67 eV, and A_1_(LO) exhibits resonance at 2.67 eV.^62^ The different responses of the SL and reference NW to different excitation wavelengths also are a strong indication of the modification of the electronic band structure in an SL as compared to the constituent materials. In order to further understand the effect of SL periodicity on the electronic band structure, we performed density functional theory (DFT) calculations with the projector augmented wave method^63^ and the local density approximation (LDA) of three GaAs/GaP SLs with different periods. Local and semilocal approximations to the exchange-correlation energy, such as the LDA, are known to underestimate electronic bandgaps. However, in a previous work, Giorgi et al.^64^ performed *G*_0_*W*_0_ calculations, which provide bandgaps in good agreement with the experiments, obtaining a correction of the DFT-LDA bandgap of WZ bulk GaAs and GaP of ∼1 eV (0.91 and 0.96 eV). Therefore, as a first approximation, we assume a similar correction for the bandgap of GaAs/GaP SLs as well. The results of our calculations along with the *G*_0_*W*_0_ corrections are tabulated in Table 2. From the calculation, we find that the bandgaps of the GaAs/GaP SLs are lower than that of the bulk WZ GaAs and GaP, indicating a type II band offset. Our calculations also indicate that the bandgap decreases with increasing period. The different excitation wavelength dependence of the Raman response of the GaP reference sample and the GaAs/GaP SLs can thus be attributed to the different electronic band structures.

**Table 2:** Calculated Electronic Bandgaps of GaAs/GaP SLs of Different Periodicities

	*n*_atoms_	period (nm)	*E*_g_ LDA (eV)	*E*_g_*G*_0_*W*_0_ (eV)
WZ GaAs bulk	4		0.55	1.46^64^
WZ GaP bulk	4		1.32	2.28^64^
GaAs/GaP SL_5_	32	5.11	0.45	1.38^a^
GaAs/GaP SL_6_	40	6.39	0.43	1.36^a^
GaAs/GaP SL_10_	64	10.22	0.36	1.29^a^

a Estimates based on the average *G*_0_*W*_0_ corrections for bulk GaAs WZ and GaP WZ of Giorgi et al.^64^

## 3 Conclusions

In this work, we reported the phononic properties of GaAs/GaP SL NWs with different periods using inelastic light scattering experiments. Our results are corroborated by ab initio theoretical calculations. We were able to assign the phonon modes stemming from the SL, associating their frequencies with the atomic species involved in the vibration. We also showed the tunability of the phononic spectrum by studying the dependence of both acoustic and optical phonon modes as a function of the SL period. Namely, the number of phonon modes increases with the SL period, which results from an increased number of atoms per unit cell. The investigation of the Raman response to different excitation wavelengths of SL compared to the reference system hints at a different electronic band structure, which is also expected to be SL-period-dependent. Hence, we demonstrated the possibility of obtaining metamaterials with designed functional properties by controlling the SL period. Our results show that NW SLs allow the creation of materials with different phonon dispersions, which in turn has implications in designing thermoelectric materials with reduced thermal conductivity. Our results also have a significant impact on creating designed materials with tunable thermal, acoustic, and optoelectronic properties.

## 4 Methods

### 4.1 Growth

The GaAs/GaP SL NWs were grown on GaAs (111)B substrates by Au-assisted Chemical Beam Epitaxy (CBE) in a Riber Compact-21 system. The metal–organic (MO) precursors used for the NW growth were triethylgallium (TEGa), tertiary butylarsine (TBAs), and tertiary butylphosphine (TBP). The GaAs (111)B substrates were first coated with a 0.1 nm thick Au film at room temperature in a thermal evaporator and then transferred to the CBE growth chamber. Prior to the start of the growth, the samples were annealed at 520 ± 5 °C under TBAs flux for 20 min in order to dewet the Au film into nanoparticles and to remove the surface oxide from the GaAs substrate. The NW growth protocol consisted of four steps. In step I, the growth of a 0.5 μm long GaAs stem was performed for 60 min at 510 ± 5 °C using MO line pressures of 0.7 and 1 Torr for TEGa and TBAs, respectively. Afterward, the temperature was ramped up to 560 ± 5 °C in 5 min to initiate the growth of the GaP segment (step II) with a direct switch of the precursor fluxes without any growth interruption. The GaP segment was grown using MO line pressures of 0.7 and 2 Torr for TEGa and TBP, respectively. In step III, the growth of GaAs and GaP alternating segments forming the SL was performed at 560 ± 5 °C using the same MO line pressures. Finally, (step IV), a 1 μm long GaP top segment was grown by keeping the growth parameters (growth temperature and MO fluxes). The growth was terminated by switching off the TEGa flux and cooling the sample under the TBP flux.

### 4.2 TEM/EDX

NW transparent to the electron beam was a dispersed carbon holey grid. HR-TEM and EDX measurements were performed using a JEOL JEM F220 microscope operated at 200 kV and equipped with an EDX spectrometer.

### 4.3 Raman Spectroscopy

For the μ-Raman experiments, we transferred single NWs onto a Si substrate with a 525 nm silicon nitride coating using a micromanipulator. The polarization-resolved Raman measurements were performed in backscattering geometry with the help of a Horiba T64000 triple spectrometer in subtractive mode with a 1.800 g/mm grating and a liquid-nitrogen-cooled CCD detector. Whenever not specified otherwise, the presented spectra were collected by using a 488 nm excitation wavelength. We used power below 120 μW to perform the Raman experiments. At room temperature, we used a 100× objective with a high NA of 0.95 for focusing the excitation laser and collecting the scattered light. We use the conventional Porto notation of the form *k_i_*(ε*_i_*, ε_s_)*k*_s_ to indicate the polarization configuration of our Raman measurements, where *k_i_*, ε*_i_*, ε_s_, and *k*_s_ are the direction of propagation of incident photon, direction of polarization of incident photon, direction of polarization of scattered photon, and direction of propagation of scattered photon, respectively. In this work, since we use backscattering geometry, we assume the incident and scattered photon wave vectors to be antiparallel and parallel to the *x* axis. Therefore, the polarization vectors will lie in the plane perpendicular to the directions of propagation, i.e., the *yz* plane. In our experiments, we consider the NW growth axis to lie along the *z* axis. For example, the configuration *x̅*(*z*,*z*)*x*, which is the main scattering geometry used in this work, represents a measurement with excitation and detection polarizations along the NW growth axis. For the sake of comparison, we have used a reference NW sample consisting of a 500 nm long GaAs segment followed by 3 μm of GaP stem.

### 4.4 Brillouin Spectroscopy

We used a 6-pass tandem Fabry Perrot interferometer, to investigate the low-frequency acoustic phonons.^55,56,65^ The measurements were performed on as-grown NWs on the GaAs substrate using a 532 nm laser at room temperature. We used a 100x objective to excite and collect the scattered light to perform the BLS measurements.

### 4.5 DFPT Calculations

The Raman susceptibility tensors were calculated within density functional perturbation theory (DFPT)^66^ with the ABINIT code^67,68^ by computing the third derivative of the total energy (twice with respect to the application of an electric field, i.e., incident and scattered light polarization vectors, and once with respect to the phonon displacement coordinates^69^). We used the LDA for the exchange-correlation energy functional and norm-conserving pseudopotentials. We used a plane wave cutoff of 37 Ha, an energy cutoff for the fine fast Fourier transform of 76 Ha, and a strict convergence criterion of the wave function residual norm of 10–22. We studied SLs with periods of 1.28, 2.56, 3.83, 5.12, and 6,39 nm, constructed by piling up unit cells of wurtzite GaAs and GaP along the [0001] crystal axis. The Brillouin zone was sampled with a grid of 8 × 8 × *Nk*-points, decreasing *N* from 3 to 1 on going from the shorter period to the longer period. Prior to the calculation of the Raman spectra, all systems were structurally relaxed, optimizing both the atomic positions and the cell lattice vectors, without applying any additional constraint. The calculations were performed in a bulk system. Further details can be found, e.g., in ref (17).

## Supporting Information Available

TEM analysis; Raman spectra in different polarization configurations; calculated phonon dispersion of SLs with different periodicity; eigen displacement of phonon modes in an SL unit cell; and analysis of BLS spectrum (PDF)
