# Supplementary material for: GaAs/GaP Superlattice Nanowires for Tailoring Phononic Properties at the Nanoscale: Implications for Thermal Engineering
Source: ACS Appl Nano Mater. 2023 Oct 5;6(19):18602–13. doi: 10.1021/acsanm.3c04245 (PMC10580287; doi:10.1021/acsanm.3c04245)
Supplement: Supplementary file 1 — an3c04245_si_001.pdf [file an3c04245_si_001.pdf]

Supporting Information

GaAs/GaP Superlattice Nanowires for Tailoring Phononic Properties at the Nanoscale: Implications for Thermal Engineering

*Aswathi K. Sivan[1], Begoña Abad[1], Tommaso Albrigi[2], Omer Arif[3], Johannes Trautvetter[1], Alicia Ruiz Caridad[1], Chaitanya Arya[1], Valentina Zannier[3], Lucia Sorba[3], Riccardo Rurali[2], and Ilaria Zardo[*1,4]*

[1]Department of Physics, University of Basel, 4056 Basel, Switzerland

[2]Institut de Ciència de Materials de Barcelona, ICMAB-CSIC, Campus UAB, 08193 Bellaterra, Spain

[3]NEST, Istituto Nanoscienze-CNR and Scuola Normale Superiore, 56127 Pisa, Italy

[4]Swiss Nanoscience Institute, University of Basel, 4056 Basel, Switzerland

*E-mail: ilaria.zardo@unibas.ch

Supporting Information 1: HR-TEM of GaAs/GaP SL nanowires

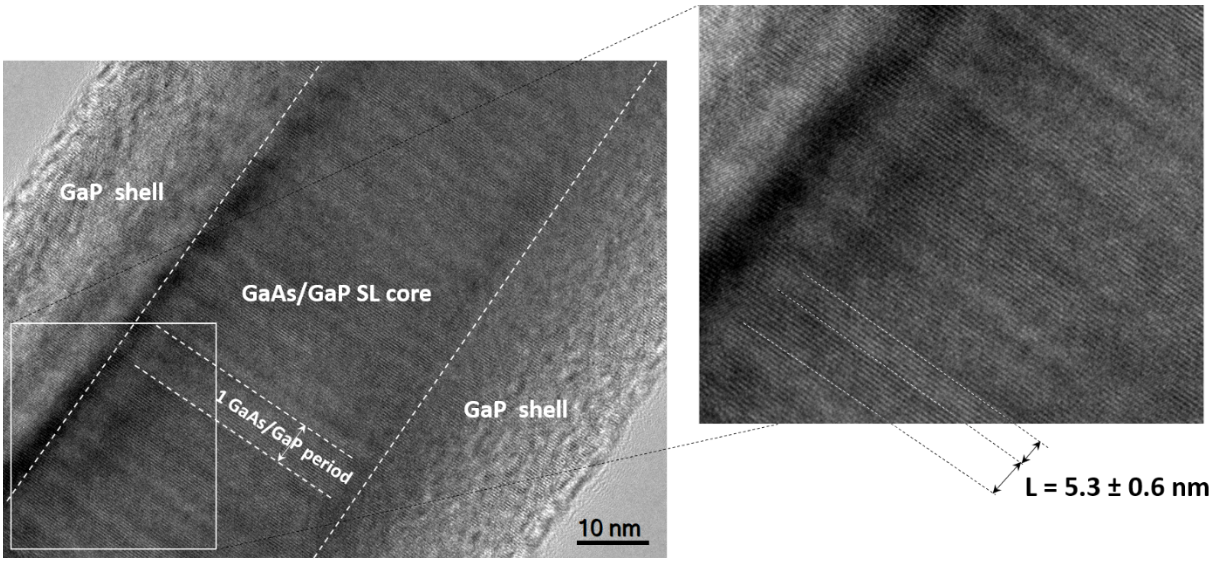

Figure S1. HR-TEM of a GaAs/GaP SL NW with nominal period of 4.8 nm. The inset zooms in on the SL segment.

In Figure S1, we show the HR-TEM of a GaAs/GaP SL NW with 4.8 nm periodicity. We used the software DigitalMicrograph^{TM} on the HR-TEM and measured a period of 5.3 ± 0.6 nm, which is in good agreement with the nominal period (4.8 nm) determined through the growth protocol.

Supporting information 2: Polarization dependent Raman scattering experiments

In figure S2(a), we present results on the μ-Raman measurements in the $\bar{x}(y,y)x$ scattering configuration on the reference NW. For comparison we also present the theoretically calculated spectra of WZ GaP in figure S2(b).

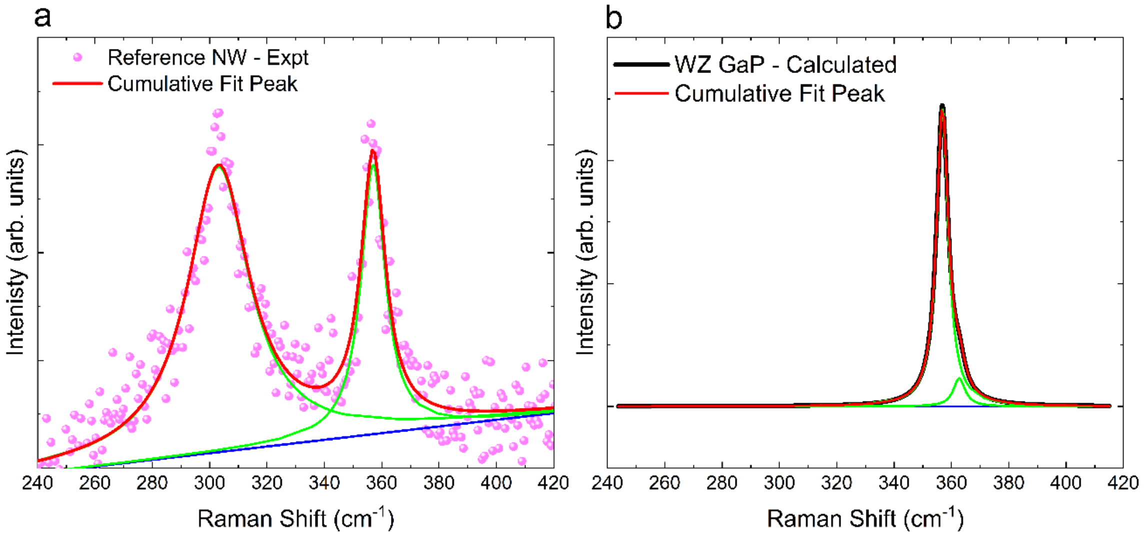

Figure S2. (a) Polarized μ-Raman spectrum on a reference NW collected in the $\bar{x}(y,y)x$ scattering configuration. The experimental data are shown in pink spheres, light green curves show the individual Lorentzian fitting, the red curve shows the cumulative fitting and dark blue line is the baseline. (b) Theoretically calculated spectrum of WZ GaP in black curve, with the full width at half maximum fixed at 5 cm^{-1}. The green curves show the individual Lorentzian fitting and the red curve shows the cumulative fitting. The dark blue line is the baseline.

In Figure S2(a), we see a peak at 356.9 cm^{-1} which can be attributed to the E_2^H of WZ GaP[1]. There is another peak at 303 cm^{-1}, which is due to the silicon substrate[2]. In Figure S2(b), we present the calculated spectrum of WZ GaP. The spectrum has two peaks at 356.8 cm^{-1} and at 362.7 cm^{-1}, which can be attributed to the E_2^H and TO of GaP, respectively. The presence of E_2^H mode confirms the WZ crystal phase of the NWs. In the experimental spectrum, in the $\bar{x}(y,y)x$ configuration, the resolution of the TO mode difficult which is why it is not used in the fitting in Figure S2 (a).

In Figure S3 (a), we present the results on the μ-Raman measurements in the $\bar{x}(y,y)x$ and $\bar{x}(z,z)x$ on SL NW with a period of 10 nm. In the $\bar{x}(z,z)x$ scattering configuration, the most intense peak in the GaAs-like phonon modes region is at 276.9 cm^{-1} while in the GaP-like phonon modes region there is an intense peak at 357.6 cm^{-1}. In the $\bar{x}(y,y)x$ scattering configuration, the most intense peak in the GaAs-like phonon modes region is at 269.4 cm^{-1} while in the GaP-like phonon modes region there is an intense peak at 351.6 cm-1. The overall

intensity of the spectrum decreases in the $\bar{x}(y, y)x$ as compared to $\bar{x}(z, z)x$ configuration by a factor of about 2, possibly due also to the dielectric mismatch effect.[3] In Figure S3 (b), we show the calculated Raman spectrum of GaAs/GaP SL with L=6.39 nm in both the polarization configuration. The intensities are normalized for ease of comparison.

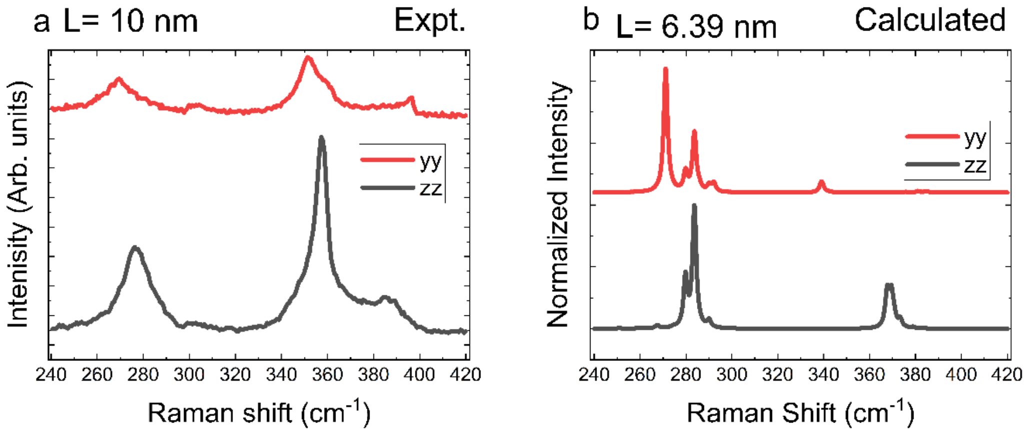

Figure S3. (a) Polarization resolved µ-Raman spectra of a 10 nm SL NW. The red curve shows the data collected in the $\bar{x}(y, y)x$ configuration and the black curve shows the data collected in the $\bar{x}(z, z)x$; (b) Calculated Raman spectra of a GaAs/GaP SL of L=6.39 nm. The intensities are normalized. The red curve shows the $\bar{x}(y, y)x$ configuration and black curves show the $\bar{x}(z, z)x$.

In Table S1 we list the computed Γ-point frequencies, setting a threshold intensity indicative of their experimental detectivity.

Table S1. The calculated frequencies of Raman active phonon modes for a GaAs/GaP SL structure with a period of 5.12 nm. Phonon modes with a Raman intensity more than 100 times smaller than the one of the most intense modes were considered non-detectable.

Frequency (cm^{-1}) (Calculated for 5.12 nm period)	Ratio of Intensity/Highest Intensity (Calculated for 5.12 nm period)	Frequency (cm^{-1}) (Experimental for 4.8 nm period)	Ratio of Intensity/Highest Intensity (Experimental for 4.8 nm period)
245.55	5E-03	261.3	8E-02
254.87	1E-02	276.4	9E-01
264.73	1E-02	287.6	5E-02
273.24	9E-02		
281.13	7E-02		
282.45	1		
288.12	1E-01		
367.25	2E-01	352.8	7E-01
368.09	1E-01	358.3	1
370.23	2E-01	368.1	8E -02
372.06	2E-02	376.7	7E-02
375.85	3E-02	388.2	1E-01
382.31	3E-03	391.98	2E-01
		394.7	1E-01

Supporting information 3: Phonon dispersion of GaAs/GaP SL with a period of 6.39 nm

The phonon dispersion of the GaP/GaAs SL with period 6.39 nm obtained through *ab initio* calculations is displayed in Figure S4. The increased number of phonon modes at the Γ-point can be appreciated.

Ab initio calculations provide us also with the relaxed lattice parameters of the constituents of the superlattice. We obtain lattice parameters $a = 3.947$ and $c = 6.509$ Å for wurtzite GaAs and $a = 3.796$ and $c = 6.262$ Å for wurtzite GaP. Since in all the SLs investigated the length of GaAs and GaP segments is very close and the elastic constants of the constituent materials are similar, the lattice parameter, a, of all the cases studied is roughly the average of the bulk cases, i.e. 3.871 Å. The c lattice parameter, on the other hand, must accommodate the period of each SL and is $12.775, 38.328$, 51.103, and 63.880 Å. Neglecting the chemical identity in all SLs, we would obtain an "effective" c lattice parameter of 6.388 Å that, again, is essentially the average of the values of wurtzite GaAs and wurtzite GaP.

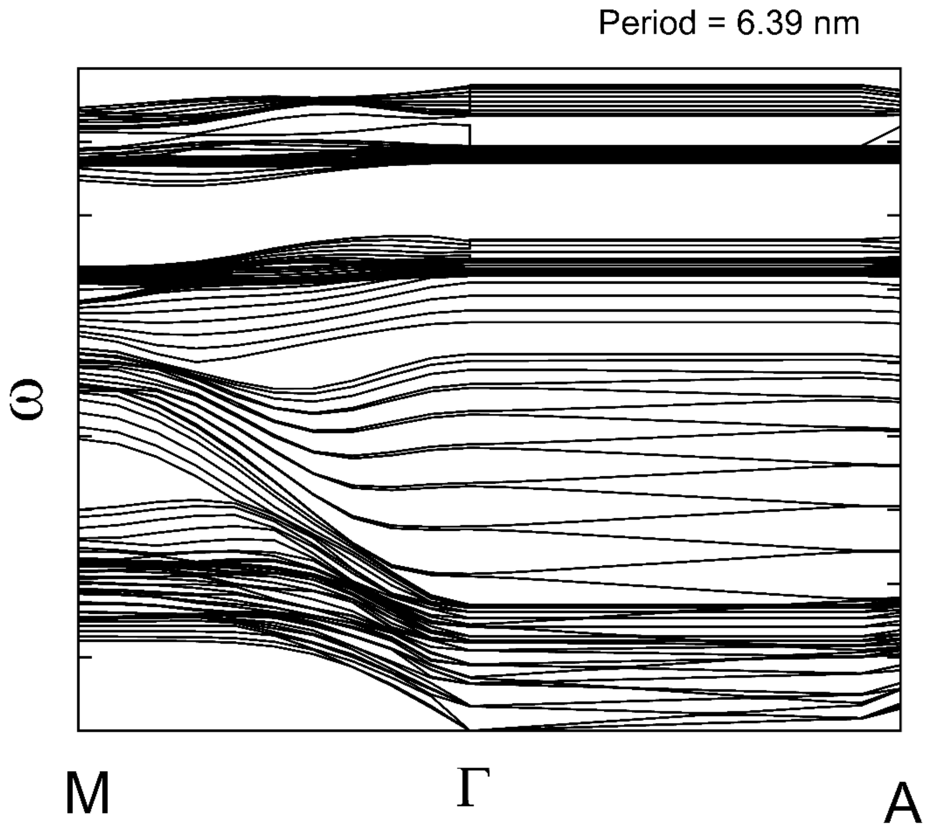

Figure S4. Calculated phonon dispersion of a GaAs/GaP SL NW with period 6.39 nm.

Table S2. Calculated acoustic phonon mode frequencies with frequencies less than 1 THz for peaks corresponding to q value close to 85 μm^{-1} from the dispersion relation in Figure S3 for SL with periodicity of 6.3 nm.

Period	Frequency (GHz)
6.3 nm	33
	74
	444
	476
	814
	835
	877

Supporting information 4: Eigen displacement of phonon modes involving different atoms in the SL unit cell

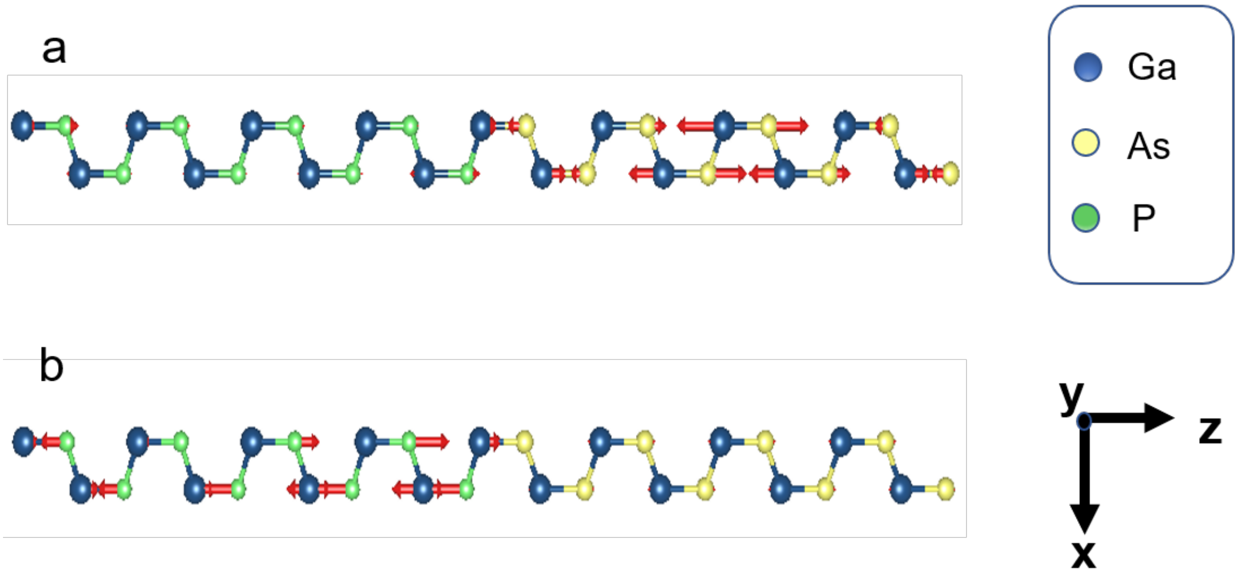

Figure S5. The eigen-displacement of atoms for phonon mode at (a) 288.12 cm^{-1} and (b) 367.25 cm^{-1}. The different colored spheres represent the constituent atoms with blue as Ga, yellow as As, and green as P.

In Figure S5, show the schematic for two phonon modes at 288.1 cm^{-1} and 367.2 cm^{-1}, respectively, selected from Table S1. The Ga atoms are represented by blue spheres, As by yellow spheres, and P with be green spheres.

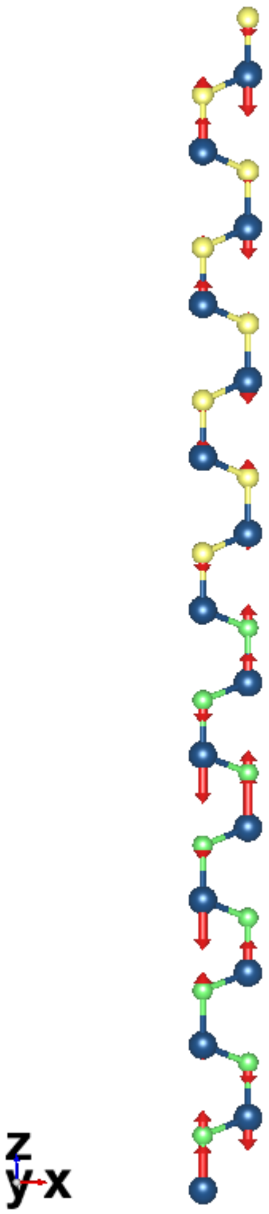

Figure S6. The Eigen displacement of atoms for phonon mode at 218 cm^{-1}. The different coloured spheres represent the constituent atoms with blue as Ga, yellow as As, and green as P.

In Figure S6, we show the eigen displacement of the atoms of the Raman inactive phonon mode at 218 cm^{-1}, whose vibrations involve all atoms in the SL unit cell. The Ga atoms are represented by blue spheres, As by yellow spheres, and P will be green spheres.

Supporting information 4: Analysis of Brillouin light scattering interferometry

In Figure S7, we present an example of data analysis of BLS spectrum. The data are fitted using deconvoluted Lorentzian curves as shown as green solid lines in the Figure. The cumulative fit is shown as red solid line.

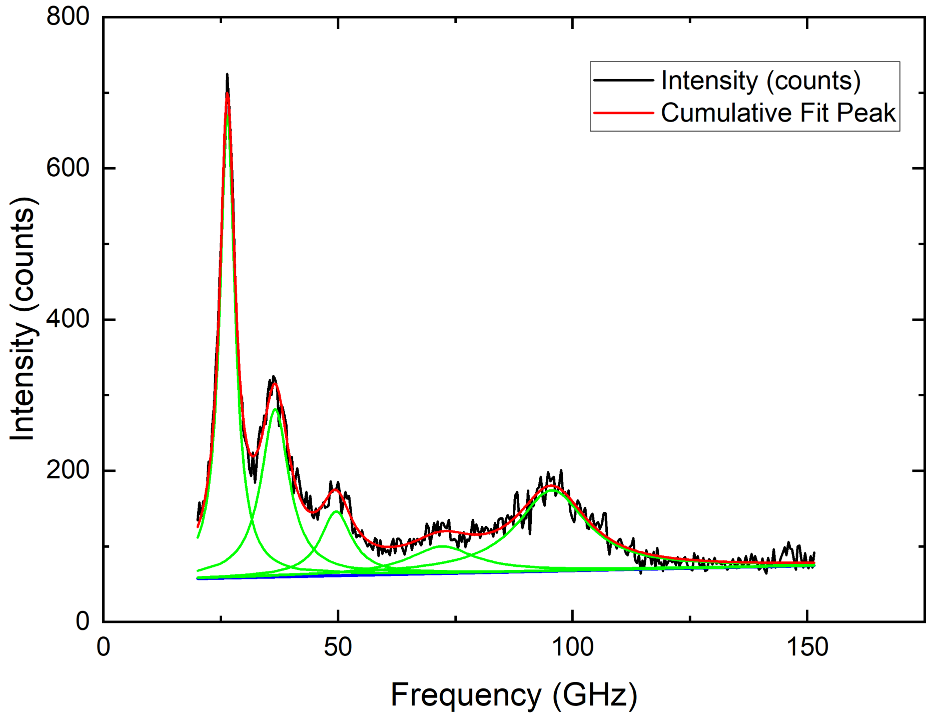

Figure S7. An example of BLS spectrum analysis. An example of deconvolution for a SL NW with period 6 nm. To analyse the data, we used peak deconvolution with several Lorentzian functions, shown in green curves. The black curve is the experimental data and the red curve is cumulative fit.

References

(1) Gupta, R.; Xiong, Q.; Mahan, G. D.; Eklund, P. C. Surface Optical Phonons in Gallium Phosphide Nanowires. *Nano Lett.* **2003**, *3* (12), 1745–1750.

(2) Jia, X.; Lin, Z.; Zhang, T.; Puthen-Veettil, B.; Yang, T.; Nomoto, K.; Ding, J.; Conibeer, G.; Perez-Wurfl, I. Accurate Analysis of the Size Distribution and Crystallinity of Boron Doped Si Nanocrystals via Raman and PL Spectra. *RSC Adv.* **2017**, *7* (54), 34244–34250.

(3) Zardo, I.; Conesa-Boj, S.; Peiro, F.; Morante, J. R.; Arbiol, J.; Uccelli, E.; Abstreiter, G.; Fontcuberta i Morral, A. Raman Spectroscopy of Wurtzite and Zinc-Blende GaAs Nanowires: Polarization Dependence, Selection Rules, and Strain Effects. *Phys. Rev. B* **2009**, *80* (24), 245324.
